# Discovery of Biomarkers for Myogenous Temporomandibular Disorders Through Salivary Metabolomic Profiling: A Pilot Study

**DOI:** 10.11607/ofph.3353

**Published:** 2023-09-30

**Authors:** Shing Ching Khoo, Yoshinobu Shoji, Chin Hoe Teh, Nyuk Ling Ma

**Affiliations:** ^1^BIOSES Research Interest Group, Faculty of Science and Marine Environment, Universiti Malaysia, Terengganu, Malaysia; ^2^Center for Oral and Maxillofacial Diagnostics and Medicine Studies, Faculty of Dentistry, Universiti Teknologi MARA, Selangor, Malaysia; ^3^Bruker Malaysia Sdn Bhd, Pulau Pinang, Malaysia

**Keywords:** Temporomandibular disorders, Salivary diagnosis, NMR analysis, Metabolomics

## Abstract

**Aims:** To develop a new approach to provide insights into contributing factors to the etiology and pathogenesis of temporomandibular disorders (TMDs) through discrimination of the salivary metabolomic profiling of patients with TMDs of muscular origin (*i.e.*, local myalgia) and healthy individuals. **Methods:** Saliva samples from 19 patients with TMDs of muscular origin (*i.e.*, local myalgia) and 39 healthy controls were collected and identified by nuclear magnetic resonance (NMR) spectroscopy. 1H NMR spectra for all samples were acquired using a Bruker Avance-III NMR spectrometer operating at 500 MHz, and data processing was performed in TopSpin, MestreNova, SIMCA, and AMIX softwares for metabolite identification. **Results:** Eight key metabolites were identified between the healthy controls and patients: L-isoleucine, methylmalonic acid, isopropanolamine, dimethyl sulfone, lactic acid, 4-ethoxyphenylacetic acid, N-acetyl alanine, and D-galactose. **Conclusions:** The results of this study demonstrate that NMR-based metabolomics coupled with multivariate data analysis is a powerful method for the metabolomic profiling of patients with TMDs of muscular origin (*i.e.*, local myalgia).

Temporomandibular disorders (TMDs) are a heterogenous subgroup of craniofacial pain problems that involve the temporomandibular joint (TMJ), masticatory muscles, and associated head and neck musculoskeletal structures [1]. TMDs have been identified as a major cause of nonodontogenic pain in the orofacial region and are considered a subclassification of musculoskeletal disorders [2]. The prevalence of TMDs has been reported to be between 10% and 26% of the US population, with an estimated annual treatment cost of $32 billion [3]. TMD diagnosis is often complicated because of the complex interplay between the central and peripheral nervous systems, cortical processing, and endogenous modulation. The cause of TMDs is usually multifactorial and is not confined to single etiologic factors. The common etiologic factors of TMDs include degenerative, inflammatory, traumatic, and genetic disorders and behavioral factors [4, 5].

Regardless of the etiology, these disorders are manageable if diagnosed at an early stage [1, 6]. Unfortunately, the diagnosis of these conditions frequently entails costly clinical and imaging tests or invasive surgical procedures that can only be performed by well-trained professionals [7]. In addition, TMD symptoms were intensified under the aggravation of psychoemotional factors caused by the coronavirus pandemic [8].

Unbiased metabolomic profiling has rapidly enhanced disease characterization and biomarker discovery. Saliva, an oral fluid that contains proteins, metabolites, and genetic molecules, offers distinctive advantages over other body fluids because it can be collected noninvasively by individuals with modest training [9, 10, 11]. Thus, unbiased metabolomic profiling of human saliva may offer an attractive alternative strategy for biomarker discovery in patients with TMDs.

To fully empower salivary diagnostics to become an approach for TMDs, we proposed to identify salivary biomarkers for screening and predicting the early onset of TMDs and/or for evaluating disease activity using a system biology approach (*i.e.*, metabolomics). This study aimed to assess the global metabolic changes in saliva underlying myogenous TMDs and to identify key metabolites as useful biomarkers for future studies. To date, only one group of Brazilian researchers has attempted to link salivary biomarkers to TMDs; therefore, it is critical to compare biomarkers from different ethnic backgrounds [12].

## Materials and Methods

### Patients

Nineteen patients with localized myalgia who had been referred to the Dental Centre, Universiti Teknologi MARA (UiTM), Selangor, Malaysia, participated in the study. These patients (5 men, 14 women) were aged 17 to 56 (median = 26) years and had localized myalgia. In addition, 39 healthy UiTM students (6 men and 33 women) aged 20 to 25 (median = 21) years who underwent the same clinical evaluation to exclude the presence of TMDs were included as controls (Table 1). The case and control groups were sex-matched (*p* < 0.05, *z*-test) but not age-matched (*p* > 0.05, Student *t* test) because of the demographics of the patients visiting the UiTM Dental Centre. All patients were diagnosed by a trained orofacial pain specialist who made a clinical judgment that the pain was primarily of muscular origin (Y.S.) [13]. All patients had a complete medical and dental history. History-taking investigated pain, limitation of mouth opening, and TMJ sounds. The clinical examination, which was performed according to the Diagnostic Criteria for Temporomandibular Disorders (DC/TMD) [13], included evaluation of pain and tenderness upon palpation of the masticatory muscles and TMJs and mandibular range of motion. The degrees of behavioral, psychologic, and psychosocial distress were evaluated using the General Health Questionnaire (GHQ-12) [14]. The scores ranged from 0 to 12, with a higher score indicating a higher level of distress.

**Table 1.** Demographic Data of Subjects.

Type	Control	TMD
Number	39	19
Mean (min–max) age, y	21 (20–25)	26 (17–56)
Sex	6 male, 33 female	5 male, 14 female

The inclusion criterion was patients with local myalgia according to the Diagnostic Criteria for TMD (DC/TMD) Axis I [13]. The exclusion criteria were patients with arthralgia, disc displacement with and without reduction, degenerative joint disease, subluxation, dental pain, neuropathic pain, a history of liver or kidney dysfunction, symptoms of acute illness (*i.e.*, fever, sore throat, body aches, diarrhea), intake of medications with anticholinergic actions (*e.g.*, tricyclic antidepressants, antipsychotics) within 48 hours of the investigation, and visible oral lesions at the time of enrollment.

In addition, patients with GHQ-12 scores with more than four indicators of probable nonpsychotic psychiatric disorders [14] were excluded. Pain intensity was not evaluated. The study was approved by the UiTM Institutional Review Board, 600-IRMI (5/1/6), REC/100/16, and all participants provided written informed consent as part of the study protocol.

### Collection of Saliva and Sample Preparation

Saliva collection was performed following the protocols of previous studies on the diagnostic applications of saliva [9, 10, 11, 12]. The timing of collection was standardized as much as possible to be between 9:00 and 11:00 am. The participants were instructed not to ingest food or drink (except water) that morning before collection and were asked to rinse the mouth with 40 mL of distilled water prior to collection. Then, saliva samples (5 mL) were collected into sterilized universal tubes. Saliva was collected by the same specialist who made the clinical judgment diagnoses.

All samples were maintained on ice and stored at −80 °C until use. For the acquisition of salivary nuclear magnetic resonance (NMR) data, the frozen harvested saliva samples were thawed, and 1-mL aliquots were spun at 3000 rpm to remove particulate matter. All saliva samples were prepared by adding deuterated phosphate buffer (pH 7.4) with sodium 3-trimethylsilyl-(2,2,3,3-2H4)-1-propionate (TSP-d_4_) as an internal standard; the deuterated solvent served as a field frequency lock. Sodium azide was added for biologic stabilization.

### 1H NMR Metabolomic Profiling

All samples were analyzed using an NMR spectrometer (Avance-III, Bruker) equipped with a broad band fluorine observation (BBFO) room temperature probe operating at a 500-MHz 1H observation frequency using the NOESYGPPR1D parameter. All NMR spectra were phased, baseline corrected, and manually referenced to the TSP-d_4_ peak at 0.0 ppm using TopSpin 3.1 software (Bruker).

### Data Processing and Analysis

The sample size needed was calculated using the MetSizeR method where the NMR data are divided into spectra bins (representing variables), and the signal intensities within the bins represent the abundance of metabolites. According to Billoir *et al*. in 2015 [15], for biomarker discovery, a sample size of 20 is sufficient for metabolic phenotypes and biomarker analysis.

The collection of spectra from all samples was processed with baseline correction, phase correction, and reference alignment using MestreNova version 14.1.0 (Mestrelab Research). The overall spectra were analyzed within 0.15 to 8.5 ppm. The spectral region from δ −0.02 to +0.02, which corresponded to TSP-d_4_, was excluded. The spectra ranging from δ 4.68 to 4.95 were cut out because they corresponded to a residual water signal, and the spectra ranging from δ 5.5 to 6.8, which corresponded to the region without any peaks observed in the stack plot, were also eliminated to minimize the noise variation. The total spectral area was calculated for the remaining bins, and normalization of the total area was carried out on the data before pattern recognition.

A stacked plot consisting of 58 samples was created, and the digitized data were exported into an Excel file before being loaded into SIMCA software (Sartorius) for principal component analysis (PCA). Furthermore, the negative values in the bucket table were replaced with 0 to avoid affecting the results of the statistical analysis. The data were first examined in SIMCA with all scaling options, including none, unit variance (UV), Pareto (Par), and centering (Ctr). The optimum scaling method was chosen based on the highest R^2^ and Q^2^ values for the same number of component comparisons. For the PCA model, R^2^ (goodness of ﬁt) was used to explain variation in the data. The ability of the model to predict the proportion of variance was assessed using Q^2^ (goodness of prediction). Projection to latent structure discriminant analysis (PLS-DA) with UV-scaled spectral data was also carried out to improve the classification of the different groups of individuals and to optimize the identification of changes that were unique to a particular group.

### Metabolite Identification

The variable of importance in the projection (VIP) plot extracted from the PLS-DA model was used to check the differentiating bins and finally used for metabolite detection. Metabolite identification was performed using AMIX 4.0.2 software (Bruker) by adopting the profiling database BBIOREFCODE 2.0. The analysis was performed on the bucket table, which was highlighted by the VIP and manual spectral comparison from the database. The identification of all primary metabolites was performed through comparison to the human salivaryrelated literature, the Human Metabolome Database, and the Biological Magnetic Resonance Data Bank.

## Results

### Salivary Metabolite Profile of 500-MHz 1H NMR Spectra

Typical 500-MHz 1H NMR spectra of saliva samples were obtained from individuals in two different groups: healthy controls (C) and patients (P) with TMDs of muscular origin (*i.e.*, local myalgia). The highquality and well-resolved NMR spectra contained peaks from a wide range of low–molecular-weight metabolites with diverse classes of organic compounds, such as amino acids, organic acids, monosaccharides, quaternary ammonium salts, and glycoproteins. A stack plot of the overlaid spectra of the 58 analyzed samples is shown in Fig. 1.

**Fig. 1.** Overlaid spectra of the 39 healthy controls (C1 to C39) and 19 TMD patients (P40 to P58) at δ 0.7 to 8.5 ppm.

### Multivariate Data Analysis of NMR Spectral Data

A comparison of all samples was first performed using unsupervised PCA to obtain an overview of the sample discrimination. The scatter plot of all samples revealed no significant differences between C and P (Fig. 2a), but when tested with different sexes, a distinct separation cluster was observed (Fig. 2b). Saliva samples from male candidates were concentrated in quadrants 2 and 4, whereas saliva samples from female candidates were concentrated in quadrants 1 and 3. For the comparison of age groups, NMR profiling of patients aged 31 to 40 years (P3 and P12) showed distinct metabolic differences from the major cluster of samples (Fig. 2c). Further examination of saliva samples from male patients showed significant differences from the control groups (Fig. 2d). Based on the PCA scatter plot (Fig. 2) and Hotelling’s T^2^ plot (Fig. 3), samples C40, P3, P13, and P17 were identified as outliers and were excluded from the analysis to avoid skewing the PLS-DA model.

**Fig. 2.** PCA score scatter plot generated from the comparison of all samples where n = 58, R^2^ = 0.979, and Q^2^ = 0.88. (a) Comparison of C *vs.* P group, (b) sex, (c) age, and (d) the four discrimination groups: control female (CF), control male (CM), patient female (PF), and patient male (PM).

**Fig. 3.** Hotelling’s T2 plot confirmed the PCA results, indicating that four samples (C40, P3, P13, and P17) were strong outliers. The dashed lines indicate 95% and 99% CI (lower and upper lines, respectively).

As women have a two times greater risk of developing TMDs than men [16], a comparison among female samples (n = 45) consisting of 13 patients and 32 controls was conducted with PCA and gave rise to a valid 3-component model with R^2^ = 0.971 and Q^2^ = 0.956 (Fig. 4a). No significant differences were observed; hence, supervised discrimination was tested with R^2^X = 0.324, R^2^Y = 0.847, and Q^2^ = 0.306 (Fig. 4b). The PLS-DA results confirmed that there were significant variations in the metabolite makeup between the C and P groups with TMDs. The data were then processed using the VIP to visualize the top 20 influencing metabolites that contributed to the changes (Fig. 4c).

**Fig. 4.** Comparison of data within the sample of female subjects. (a) Unsupervised PCA of C *vs.* P (R^2^ = 0.971, Q^2^ = 0.956). (b) Supervised PLS-DA score scatter plot of C *vs.* P. (c) VIP plot based on the PLS-DA model of P and C indicating the significance of NMR peak intensities in a descending manner. VIP value > 1.0 was remarked as a statistically significant threshold.

Potential TMD biomarkers were identified based on analysis of the top 20 spectral splitting patterns obtained from the VIP plot (see Fig. 4c). This led to the identification of eight key metabolites that could potentially be used as biomarkers for TMDs of muscular origin: These were L-isoleucine, methylmalonic acid, isopropanolamine, dimethyl sulfone, lactic acid, 4-ethoxyohenylacetic acid, N-acetyl alanine, and D-galactose (Table 2). The upregulation and downregulation of potential metabolites were obtained from the S-plot generated from the orthogonal partial least squares discriminant analysis of the same dataset (Fig. 5a). In addition, the potential metabolites showed different metabolite makeups in C and P variations (Fig. 5b).

**Table 2.** Summary of Significantly Changed Metabolites in P and C Groups.

No	Potential metabolite detected	Chemical shift	Multiplicity	Difference	Formula and structure
1.	L-isoleucine	1.03 1.16	γ-CH3 (d) β-CH3 (m)	🡫	C_6_H_13_NO_2_ 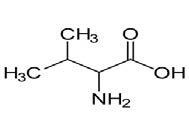
2.	Isopropanolamine	1.28 4.04	CH_3_ (s) CH (m)	🡫	C_3_H_9_NO 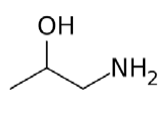
3.	Methylmalonic acid	1.28	CH_3_ (s)	🡫	C_4_H_6_O_4_ 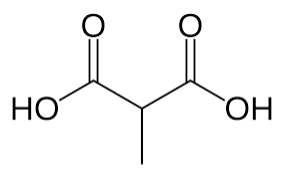
4.	Dimethyl sulfone	3.16	S-CH_3_ (s)	🡩	C_2_H_6_O_2_S 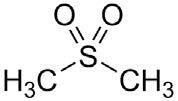
5.	Lactic acid	1.33 4.16	CH_3_(d) CH (q)	🡫	C_3_H_6_O_3_ 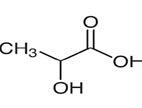
6.	4-ethoxyphenylacetic acid	4.16	Aromatic-O-CH_2_ (q)	🡫	C_10_H_12_O_3_ 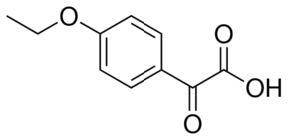
7.	N-acetyl alanine	4.20	C = N-H (q)	🡫	C_5_H_9_NO_3_ 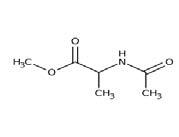
8.	D-galactose	4.55	Aromatic-O-H (s)	🡫	C_6_H_12_O_6_ 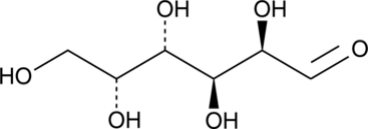

*Notes: 🡫 = decreased in TMD patients, 🡩 = increased in TMD patients. The multiplicity of chemical bonds symptom with (s) = singlet, (d) = doublet, (q) = quartet, and (m) = multiplet.*

**Fig. 5.** (a) S-plot of the selected potential biomarkers generated from PLS-DA with UV scaling. Postivie values denote upregulation, and negative values denote downregulation. (b) Representative 1H NMR spectra show the changes of metabolomic make-up in the C (bottom) and P (top) subjects. The identified key metabolites that showed distinct variations among the C and P groups (n = 58) are as follow: (1) L-isoleucine, (2) methylmalonic acid, (3) isopropanolamine, (4) dimethyl sulfone, (5) lactic acid, (6) 4-ethoxyohenylacetic acid, (7) N-acetyl alanine, and (8) D-galactose.

## Discussion

There is always a major statistical challenge when discriminating a high degree of interdependency molecules within a biologic system because of the high complexity and discrepancies between the number of study objects and number of variables analyzed [17]. Therefore, further in-depth accentuation for statistical analysis is needed when analyzing mass complex data involving RNA, proteins, and metabolites. Multivariate statistical analysis has been an advanced tool employed to study metabolomic changes and potential biomarker detection in clinical and therapeutic applications [18]. Geng *et al*. [19] revealed that a VIP value of > 1.0 was a key parameter and served as a significant statistical threshold to reveal the differences in metabolites between study participants.

The eight important metabolites identified in this study could serve as biomarkers for TMD identification in healthy controls and patients with TMDs of muscular origin (*i.e.*, local myalgia). These metabolites were L-isoleucine, methylmalonic acid, isopropanolamine, dimethyl sulfone, lactic acid, 4-ethoxyohenylacetic acid, N-acetyl alanine, and D-galactose.

L-isoleucine is a functional amino acid that plays a crucial role in the energy supply for muscle tissue and enhances muscle growth [20]. When a patient has TMD pain, there is a possibility that the L-isoleucine–maintained muscle growth has been depleted. As mentioned by Kanehira *et al*. [21] and Salameh *et al*. [22], physiologic stress and depression have been implicated as risk indicators for TMDs. The potential of L-isoleucine as a biomarker is in agreement with Solís-Ortiz *et al*. [23], who revealed that deficiency in L-isoleucine contributed to a higher risk of depression, especially in women. Methylmalonic acid (MMA) is one of several acids present in urine and plasma, and isopropanolamine is an amino alcohol [24], but to the best of our knowledge, there are no studies that have shown an association between these substances and TMD pain.

Dimethyl sulfone, also known as methylsulfonylmethane, has shown excellent anti-inflammatory mechanisms against TMJ disorders [25]. Methylsulfonylmethane demonstrated an excellent inhibitory effect on NF-κB, which significantly impacts the reduction of tumor necrosis factors (TNFs) such as TNF-α [26], as well as cytokine mediators such as IL-6 and IL-1β, which are greatly associated with TMD pain [27]. Lactic acid has historically been considered the cause of delayed-onset muscle soreness (DOMS) because of its high production rates during exercise. A study by Schwane *et al*. [28] measured blood lactic acid concentration before and during two different 45-minute treadmill exercises, one on a level surface and one at a 10% decline, and found that DOMS was not prevalent in level-surface runners even though the lactic acid concentration was significantly increased. Conversely, downhill runners saw no significant increase in lactic acid concentrations but experienced significant DOMS [28]. It now appears that increased lactate production and concentration as a result of anoxia or dysoxia (hypoxia) are often the exceptions rather than the rule. Lactate can no longer be considered the usual suspect for metabolic “crimes” but is instead a central player in cellular, regional, and whole-body metabolism [29]. 4-methoxyphenylacetic acid is a monocarboxylic acid, N-acetyl alanine is a product of the enzyme, and D-galactose is a monosaccharide sugar [24], but there are no studies that have shown an association between these substances and TMD pain.

Several types of biomarkers have been reported for TMD diagnosis, including (1) genetic biomarkers, such as serotonin receptor, muscle RAS oncogene homolog, glucocorticoid receptor, and catechol-O-methyltransferase; (2) molecular biomarkers involved in neuronal signaling molecules, such as calcitonin gene-related peptide and dopamine; (3) cytokine and inflammatory mediators, such as IL-6, IL-1β, and TNF-α; (4) neuroradiologic biomarkers, such as the promising neurobiologic imaging technique including functional magnetic resonance imaging and diffusion tensor imaging allowing better quantification of psychologic, structural, cognitive, and chemical changes that occur in chronic TMD pain; and (5) psychophysical biomarkers, such as observed sensory abnormalities and enhanced pain sensitivity [30]. However, the identification of metabolites as biomarkers is currently scarce, and there is a large knowledge gap that has to be studied and investigated further.

To our knowledge, there has only been one such literature report on salivary biomarkers in patients with TMDs so far [12]. Moreover, recent work that demonstrated a lower level of human herpes virus 6 viral genome in the saliva of patients with TMDs of muscular origin (*i.e.*, local myalgia) than in the saliva of healthy individuals implicates the possibility of an infectious basis of TMD pathogenesis and further suggests the potential of using salivary biomarkers for the diagnosis of this complex disease [31]. The common features of TMDs are that ideal management is possible if diagnosed at an early stage. Unfortunately, traditional clinical criteria are often insufficient for determining the sites of active disease, quantitatively monitoring the response to therapy, and measuring the degree of susceptibility to future disease progression. This is mainly due to a poor understanding of the specific pathophysiology of TMDs [32, 33]. The TMJs or masticatory muscles as superficial structures are significantly more accessible than the trigeminal nerve. Hence, the study of salivary samples for the detection of TMD biomarkers is more endearing than the collection of nerve biopsy or cerebrospinal fluid [30].

## Conclusions/Clinical Implications

By using an unbiased system biology approach to profile salivary metabolites from diseased patients and matched healthy individuals, this study is expected to provide a robust scientific platform for future development of point-of-care technologies for high throughput, efficiency, and accurate clinical implications. The most difficult aspect of salivary diagnostics is the identification of diagnostic indicators of the illness. We do not rule out the possibility that there might be a selection bias in this study due to the small sample size, non–age-matched sample, and the lack of assessment of pain intensity. The detection of metabolites in salivary samples as diagnostic indicators through NMR remains largely undiscovered, and further investigation is recommended to determine the clinical impact of potential biomarkers in the diagnosis of TMDs of muscular origin (*i.e.*, local myalgia).
